# Supplementary material for: Quantum game strategy solution for R&D cartel: Reorganizing government R&D investment strategy in Korea
Source: PLoS One. 2024 Dec 5;19(12):e0308355. doi: 10.1371/journal.pone.0308355 (PMC11620664; doi:10.1371/journal.pone.0308355)
Supplement: S1 Appendix — (DOCX) [file pone.0308355.s001.docx]

**S1 Appendix. Python code for calculations Table 4 and graphs Fig 3.**

import numpy as np

# Define matrices

I = np.array([[1, 0], [0, 1]])

sigma_x = np.array([[0, 1], [1, 0]])

sigma_y = np.array([[0, -1j], [1j, 0]])

sigma_z = np.array([[1, 0], [0, -1]])

H = (1/np.sqrt(2)) * np.array([[1, 1], [1, -1]])

# Define theta and gamma

theta = np.pi/4

gamma = np.pi/2

# Define X matrix

X = np.cos(2*theta) * I + 1j * np.sin(2*theta) * sigma_x

# Define entanglement matrix U

U = (1/np.sqrt(2)) * (np.kron(I, I) + np.exp(1j*gamma) * np.kron(X, X))

# Initial state

CC = np.array([[1], [0], [0], [0]])

# Calculate psi_1

psi_1 = np.dot(U, CC)

strategies = {

'I': I,

'sigma_x': sigma_x,

'sigma_y': sigma_y,

'sigma_z': sigma_z,

'H': H

}

# Strategy list

strategy_list = [

('I', 'I'),

('I', 'sigma_x'),

('sigma_x', 'I'),

('sigma_x', 'sigma_x'),

('I', 'H'),

('H', 'I'),

('H', 'sigma_x'),

('sigma_x', 'H'),

('H', 'H'),

('sigma_x', 'sigma_y'),

('sigma_y', 'sigma_x'),

('sigma_y', 'sigma_y'),

('sigma_x', 'sigma_z'),

('sigma_z', 'sigma_x'),

('sigma_z', 'sigma_z'),

('sigma_y', 'sigma_z'),

('sigma_z', 'sigma_y')

]

results = []

# Calculate psi_2, psi_3, and expected payoffs for each strategy

for strategy in strategy_list:

RA = strategies[strategy[0]]

RB = strategies[strategy[1]]

psi_2 = np.dot(np.kron(RA, RB), psi_1)

U_dagger = U.conj().T

psi_3 = np.dot(U_dagger, psi_2)

pi_A = 3 * abs(psi_3[0])**2 + 0 * abs(psi_3[1])**2 + 5 * abs(psi_3[2])**2 + 1 * abs(psi_3[3])**2

pi_B = 3 * abs(psi_3[0])**2 + 5 * abs(psi_3[1])**2 + 0 * abs(psi_3[2])**2 + 1 * abs(psi_3[3])**2

results.append((strategy, psi_2, psi_3, pi_A, pi_B))

results

import matplotlib.pyplot as plt

# Extract payoffs

pi_A_values = [r[3] for r in results]

pi_B_values = [r[4] for r in results]

strategies = [f"{s[0]}, {s[1]}" for s in strategy_list]

# Convert complex numbers to real numbers for plotting

pi_A_values_real = [float(np.real(val)) for val in pi_A_values]

pi_B_values_real = [float(np.real(val)) for val in pi_B_values]

# Plot

plt.figure(figsize=(12,8))

x = np.arange(len(strategies))

width = 0.35

plt.bar(x - width/2, pi_A_values_real, width, label='Player A', color='blue')

plt.bar(x + width/2, pi_B_values_real, width, label='Player B', color='red')

plt.ylabel('Expected Payoff')

plt.title('Expected Payoff by Strategy')

plt.xticks(x, strategies, rotation=90)

plt.legend()

plt.tight_layout()

plt.savefig('Fig 3.tif', format='tif')

plt.show()
