# Supplementary material for: Quantum game strategy solution for R&D cartel: Reorganizing government R&D investment strategy in Korea
Source: PLoS One. 2024 Dec 5;19(12):e0308355. doi: 10.1371/journal.pone.0308355 (PMC11620664; doi:10.1371/journal.pone.0308355)
Supplement: S2 Appendix — (DOCX) [file pone.0308355.s002.docx]

**S2 Appendix. Python code for graph Fig 4.**

from qiskit import QuantumCircuit

from qiskit.visualization import circuit_drawer

import numpy as np

# Define the quantum circuit with 2 qubits

qc = QuantumCircuit(2)

# Step 1: Initial state |00> (|CC>) is already set by default

# Step 2: Apply the entanglement operator U

theta = np.pi / 4

gamma = np.pi / 2

qc.rz(theta, 0)

qc.rz(theta, 1)

qc.cx(0, 1)

qc.p(gamma, 1) # Apply phase shift

# Step 3: Applying RA and RB (both are Identity operations, so no change)

# Step 4: Apply the inverse entanglement operator U†

qc.p(-gamma, 1) # Apply inverse phase shift

qc.cx(0, 1)

qc.rz(-theta, 0)

qc.rz(-theta, 1)

# Step 5: Measurement of the qubits

qc.measure_all()

# Save the circuit diagram to an image file

circuit_diagram = circuit_drawer(qc, output='mpl')

circuit_diagram.savefig("Fig 4.tif")

qc.draw("mpl")
