# Supplementary material for: Quantum game strategy solution for R&D cartel: Reorganizing government R&D investment strategy in Korea
Source: PLoS One. 2024 Dec 5;19(12):e0308355. doi: 10.1371/journal.pone.0308355 (PMC11620664; doi:10.1371/journal.pone.0308355)
Supplement: S3 Appendix — (DOCX) [file pone.0308355.s003.docx]

**S3 Appendix. Python code for graph Fig 5.**

# Define gamma values

gamma_values = np.arange(0, np.pi + np.pi/30, np.pi/30)

# Strategy matrices

RA = sigma_x

RB = sigma_y

pi_A_values_gamma = []

pi_B_values_gamma = []

# Calculate payoffs for each gamma value

for gamma in gamma_values:

U_gamma = (1/np.sqrt(2)) * (np.kron(I, I) + np.exp(1j*gamma) * np.kron(X, X))

psi_1_gamma = np.dot(U_gamma, CC)

psi_2_gamma = np.dot(np.kron(RA, RB), psi_1_gamma)

U_dagger_gamma = U_gamma.conj().T

psi_3_gamma = np.dot(U_dagger_gamma, psi_2_gamma)

pi_A = 3 * abs(psi_3_gamma[0])**2 + 0 * abs(psi_3_gamma[1])**2 + 5 * abs(psi_3_gamma[2])**2 + 1 * abs(psi_3_gamma[3])**2

pi_B = 3 * abs(psi_3_gamma[0])**2 + 5 * abs(psi_3_gamma[1])**2 + 0 * abs(psi_3_gamma[2])**2 + 1 * abs(psi_3_gamma[3])**2

pi_A_values_gamma.append(pi_A)

pi_B_values_gamma.append(pi_B)

# Convert to real numbers for plotting

pi_A_values_gamma_real = [float(np.real(val)) for val in pi_A_values_gamma]

pi_B_values_gamma_real = [float(np.real(val)) for val in pi_B_values_gamma]

pi_A_values_gamma_real, pi_B_values_gamma_real

# Plot

plt.figure(figsize=(12,8))

plt.plot(gamma_values, pi_A_values_gamma_real, label='Player A', color='blue', marker='o')

plt.plot(gamma_values, pi_B_values_gamma_real, label='Player B', color='red', marker='o')

plt.xlabel('Gamma Value')

plt.ylabel('Expected Payoff')

plt.title('Expected Payoff by Gamma Value')

plt.xticks(gamma_values, [f"{val:.2f}" for val in gamma_values], rotation=90)

plt.legend()

plt.tight_layout()

plt.grid(True)

plt.savefig('Fig 5.tif', format='tif')

plt.show()
