# Supplementary material for: Quantum game strategy solution for R&D cartel: Reorganizing government R&D investment strategy in Korea
Source: PLoS One. 2024 Dec 5;19(12):e0308355. doi: 10.1371/journal.pone.0308355 (PMC11620664; doi:10.1371/journal.pone.0308355)
Supplement: Attachment — Submitted filename: Response to Reviewers_final0708.docx [file pone.0308355.s004.docx]

**< Reviewer 1>**

**1-1. The abstract lacks a description of the results, and the authors should describe key findings or valuable conclusions in the abstract. In addition, the abstract should be a complete paragraph and should not be segmented.**

=>

The abstract was completely rewritten according to the reviewer's comments (pp.1-2).

**1-2. The author’s description of game theory is not comprehensive enough. I suggest the author refer to the following papers (doi: 10.1109/TCSS.2023.3324087; 10.5109/4793672). Besides, recent research points out the fact that the study of evolutionary game theory has many applications in both the social and natural sciences, and there are many recent studies pointing this, which would be useful in the introduction to make this important point, such as 10.1109/TSMC.2023.3315963; 10.1063/5.0099697. This would further improve the introduction and the outline of possible future research.**

=>

A comprehensive comparison was made using recent papers on classical game theory and quantum game theory (Table 1: New insert, pp.4-6), and the main suggestions of the recommended paper (Feng et al., 2023) were added to future research directions (p.29).

**1-3. There are some formatting mistakes in the paper. I would suggest the author proofread the paper meticulously and thoroughly. For example, the indentation of sections and subsections is inconsistent. Besides, each formula should be numbered accordingly.**

=>

The indentation for each section and the numbering of each formula have been modified.

**1-4. The strategic combination between Alice and Bod on page 4 and page 5 is duplicated, and it is suggested that the authors delete one of them.**

=>

The existing Table 2 and Table 3 were combined and unified into Table 2 (p.7).

**1-5. The figure is missing in the body of the text! Moreover, the resolution of the figures in the paper is so low that it is difficult to see the information inside the images, the author should increase the dpi of each figure and put them in the right place in the body of the text.**

=>

Existing Fig 1 was rewritten with higher resolution, and the position of the picture was relocated to fit the text (p.12). The explanatory power of the text has been improved by adding a new Figure 1(p.11).

**1-6. The results in the paper, including figures or tables, in addition to being cited in the body of the text, are also required to be analyzed accordingly, i.e., what phenomenon each figure or table shows and what conclusions can we draw from them.**

=>

Explanatory notes for each figure included in the paper (Fig 1-Fig 5) have been inserted .

**1-7. Are the strategies of the individuals in this paper determined at the beginning and do not change in the evolutionary process? If not, why is there no simulation of how strategies change in the system?**

=>

Quantum games involve simulations that identify eigenstates by applying various types of wave functions to operators and observing the resulting changes. These simulations are important in understanding the evolutionary development of strategies in quantum game theory, and were added to the text (p.5) by explaining that they are ultimately premised on simulations that allow participants to achieve optimal results through cooperation and competition .

And on page 11, Fig 1 "Concept map for quantum information strategy game" was inserted to make the concept of simulation more understandable.

**1-8. The setting of sections and subsections in the paper is a bit confusing. It is suggested that the authors set up the model and simulation results in this paper as different sections, and then subdivide the subsections for the model and results.**

=>

Quantum game model(p.3) composition and simulation results(p.13) were constructed separately.

**1-9. There are only about five references in this paper from the last 5 years and most of the authors’ references are from before 2019, which are too obsolete. The references should cite more papers from the past five years.**

=>

More than 20 latest papers were added to the previous research and conclusions.

**1-10. Some references contain errors and inconsistent formatting. It is difficult to give credit to research if even such elementary aspects of the work are not error-free. The references should be made error-free and formatted in agreement with the journal guidelines. The authors should double-check to correct these errors.**

- We checked and corrected to ensure there were no errors.

**< Reviewer 2 >**

**2-1. The use of symbols in the equations needs to be standardized, such as whether the same symbol is italicized or not, which needs to be consistent throughout the text. In addition, it is suggested to number the equations for easier understanding and reading.**

- All equations are numbered and the use of italics is kept to a minimum for consistency.

**2-2. It's not common to use " betrayal" (Page 4, Section 2.2). Instead, this field often uses "defection".**

- All have been modified to "defection".

**2-3. When calculating , 1++ i2 equal to 1? When calculating , 1 - i2 equal to 1? In other words, i2 equal to 0? The authors should explain**.

- We confirmed it was a typo. All subsequent calculation formulas were also checked and verified. Fortunately, there was no difference from the results described in the first paper.

**2-4. The figures are not clear. It is suggested to improve their clarity.**

- The picture has been re-edited for clarity.

**2-5. Some references contain errors and inconsistent formatting. It is difficult to give credit to research if even such elementary aspects of the work are not error free. The references should be made error free and formatted in agreement with the journal guidelines**

- References were re-examined and there were no formatting errors.
